# Avoidance of Hen's Egg Based on IgE Levels Should Be Avoided for Children With Hen's Egg Allergy

**DOI:** 10.3389/fped.2020.583224

**Published:** 2021-01-15

**Authors:** Yoshitsune Miyagi, Kiwako Yamamoto-Hanada, Hiroya Ogita, Tomoyuki Kiguchi, Yusuke Inuzuka, Kenji Toyokuni, Koji Nishimura, Makoto Irahara, Fumi Ishikawa, Miori Sato, Mayako Saito-Abe, Yumiko Miyaji, Shigenori Kabashima, Tatsuki Fukuie, Ichiro Nomura, Yukihiro Ohya

**Affiliations:** ^1^Allergy Center, National Center for Child Health and Development, Tokyo, Japan; ^2^Department of Pediatrics, Nakagami Hospital, Okinawa, Japan

**Keywords:** hen's egg allergy, food allergy, avoidance, IgE, sensitization, oral tolerance, oral food challenge

## Abstract

**Background:** Although hen's egg (HE) allergy was thought to be usually resolved by late childhood, majority of HE allergy patients with a high level of egg white (HEW)-specific IgE could not acquire tolerance for HE by age 8 years.

**Objective:** The aim is to investigate whether the avoidance of HE until 6 years of age increased the risk of heated HE allergy at age 6 years.

**Methods:** This was a retrospective case-control study. The HE tolerance children (*n* = 17) and children with low-dose HE reactor [a positive reaction to ≤ 4 g of heated HEW in oral food challenges (OFCs)] children (*n* = 26) were included based on the results of OFC at 6 years old. Multivariate logistic regression analysis was applied to examine the associations between HE avoidance until age 6 years and HE allergy status confirmed by OFC, adjusting the level of ovomucoid-specific IgE (OM-sIgE) during early infancy.

**Results:** A lower proportion of strict avoidance of HE was observed in the HE tolerance group than in the low-dose HE reactor group (6 vs. 46%, *p* = 0.006). OM-sIgE levels in children younger than 2 years old were significantly higher in the low-dose HE reactor group than those in the HE tolerance group (median [interquartile], 26.7 UA/mL [11.9–53.4] vs. 7.9 UA/mL [0.35–23.4]; *p* =0.024). The avoidance of HE until 6 years of age increased the risk of heated HE allergy even after adjusting OM-sIgE levels.

**Conclusions:** The long-term avoidance of HE from infancy increased the risk of heated HE allergy confirmed by OFC at age 6 years.

## Introduction

Food allergy is one of the major global children's health issues, and hen's egg (HE) is the most common allergenic food among Japanese children (1). Ohtani et al. (2) showed that the rate of tolerance for half of whole heated HE was 30% by 3 years of age, 59% by 5 years of age, and 73% at 6 years of age and levels of egg white-specific IgE (EW-sIgE) and ovomucoid-specific IgE (OM-sIgE) in the persistent HE allergy remained significantly higher than those in the HE tolerance. Although most cases of HE allergy are thought to resolve by late childhood, a previous study revealed that 89% patients with an EW-sIgE level of >50 kU/L could not acquire a tolerance for HE by age 8 years under the condition of avoidance (3, 4). Unfortunately, 57% of children who were diagnosed with HE allergy were identified based merely (solely) on HE sensitization and were treated with HE avoidance, although they had no clinical history of an allergic reaction to HE (3). It is not clear whether the avoidance of egg among children with egg allergy is better.

Du Toit et al. (5) noted that inducing oral tolerance against allergenic foods might be effective in both cases: in young infants who were not yet sensitized and in those who were already sensitized. In a previous report called the PETIT study for the HE allergy “prevention,” 80% of children were already sensitized to HE before its introduction (6). However, the results of the PETIT study demonstrated that the early introduction of very small amounts of heated HE powder at 6 months of age and onwards was effective in preventing HE allergy and inducing oral tolerance at 12 months of age. We believe that early introduction of heated HE might be effective for both preventing HE allergy and treating HE allergy against infants who already developed HE allergy. There were no data to examine the effectiveness of early introduction of heated HE for HE-allergic infants. Based on the results of several randomized control trials (RCTs) that were conducted under experimental conditions, the Cochrane systematic review by Romantsik et al. (7) reported that OIT for HE allergy was effective in inducing oral tolerance to allergenic foods. However, there was no sufficient evidence regarding the effectiveness and safety of OIT for HE-allergic infants in real-world practice. Moreover, these RCTs were limited in their safety because dose of OIT was not small and the occurrence of adverse events among the subjects was very common.

We hypothesize that HE-allergic infants who are highly sensitized to HE in infancy may not be able to achieve oral tolerance for HE by long-term avoidance based on the presence of IgE sensitization. Furthermore, we believe that strict avoidance of HE can delay oral tolerance, thereby resulting in an increased risk of persistent HE allergy. This study aimed to investigate whether the strict avoidance of HE until 6 years of age in HE-allergic children with positive HE sIgE during early in life could increase the risk of a persistent HE allergy in clinical practice.

## Methods

### Design and Participants

Our study was designed as a retrospective single-center case-controlled study using the data from the children's medical charts. This study was approved by the ethics committee of the National Center for Child Health and Development (2019-066).

**Figure 1:**
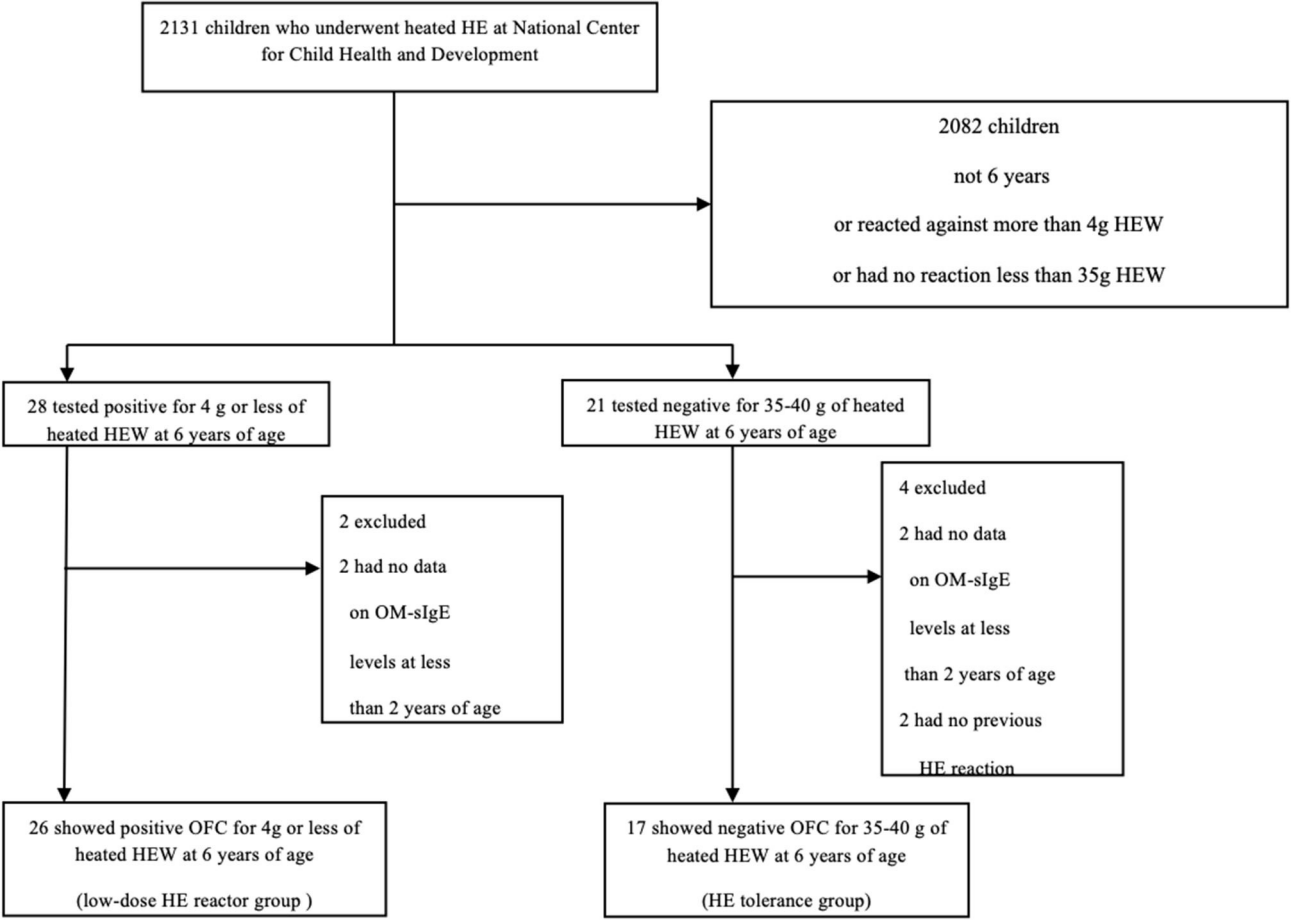
Study population.

We defined cases as OFC positive children and controls as OFC negative children at 6 years of age. Inclusion criteria were as follows: (i) sensitization to HE before 2 years of age (we measured serum concentrations of IgE specific to egg white and ovomucoid with the ImmunoCAP (Thermo Fisher Scientific, Upsala, Sweden) system and defined lower than 0.35 kUA/L as non-sensitized); (ii) children had undergone OFCs at age 6 years for heated HE white (HEW); and (iii) positive reaction to ≤ 4 g of heated HEW (low dose, equivalent to ≤ 1/10 of one HEW) or tolerance to 35–40 g of heated HEW (equivalent to ~1 HEW). We reviewed the records of children who underwent HEW OFCs at the National Center for Child Health and Development (NCCHD) between November 2013 and July 2019 and who tested positive for OM-sIgE or EW-sIgE before 2 years of age (Figure 1). Among 2131 children who underwent heated HEW OFCs at NCCHD, 49 met the inclusion criteria. After excluding children with missing data, we categorized our subjects into two groups (cases and controls). The HE control tolerance group (*n* = 17) included children who could tolerate 35–40 g of heated HEW (equivalent to ~1 heated hen's egg) in the OFCs at 6 years of age as controls, while the low-dose HE reactor group (*n* = 26) included children who showed a positive reaction to ≤ 4 g of heated HEW (low dose ≤ 1/10 of one HEW) in OFCs at 6 years of age as cases. We defined a case as children with low-dose HE reactor (those who showed allergic reactions to low-dose heated HEW) because mild or moderate HE allergy (allergic reactions to moderate-dose heated HEW) could naturally induce oral tolerance to HE. In both groups, two children (four in total) were excluded because data on OM-sIgE levels prior to 2 years of age were missing. In the low-dose HE reactor group, two children were excluded because they had no previous reactions to HE.

### OFC

We performed OFCs (8) following the Japanese Food Allergy Guidelines (9). The NCCHD conducts ~1,500 OFCs per year. The OFCs were conducted using a single dose or using a graded dosing method in 20–40 min intervals. In HE tolerance group, 14 children used 20 min-boiled egg and 3 children used scrambled egg. In low-dose HE reactor group, all children used 20 min-boiled egg. All OFCs were performed under the observation of a physician. The challenge dose was determined by the patient's physician based on ovomucoid-specific IgE levels, egg white-specific IgE levels and patient medical history. Positive reaction to OFC was defined as observational signs and/or subjective symptoms (except oral discomfort) elicited by OFC as an IgE-mediated immediate reaction.

### Variables

All the OFC data, history of avoidance, allergic comorbidity (atopic dermatitis, bronchial asthma, or allergic rhino-conjunctivitis), EW-sIgE levels, OM-sIgE levels (10), and total IgE levels were extracted from their medical chart records. Strict avoidance was defined as a zero dose of eggs, including eggs baked into cakes and biscuits.

### Statistical Analysis

For statistical analysis, the Mann Whitney-*U*-test was and the Fisher's exact test were used to analyse the variables between the two groups. Multivariate logistic regression analysis was applied to both patient groups as dependant variables, while OM-sIgE levels at <2 years of age or the peak egg white IgE levels >50 UA/ml and OIT were considered covariates (explanatory variables). A *p* < 0.05 was considered statistically significant. All statistical analyses were performed using the JMP^®^14 software (SAS Institute Inc., Cary, NC, USA, 2018).

## Result

**Figure 2:**
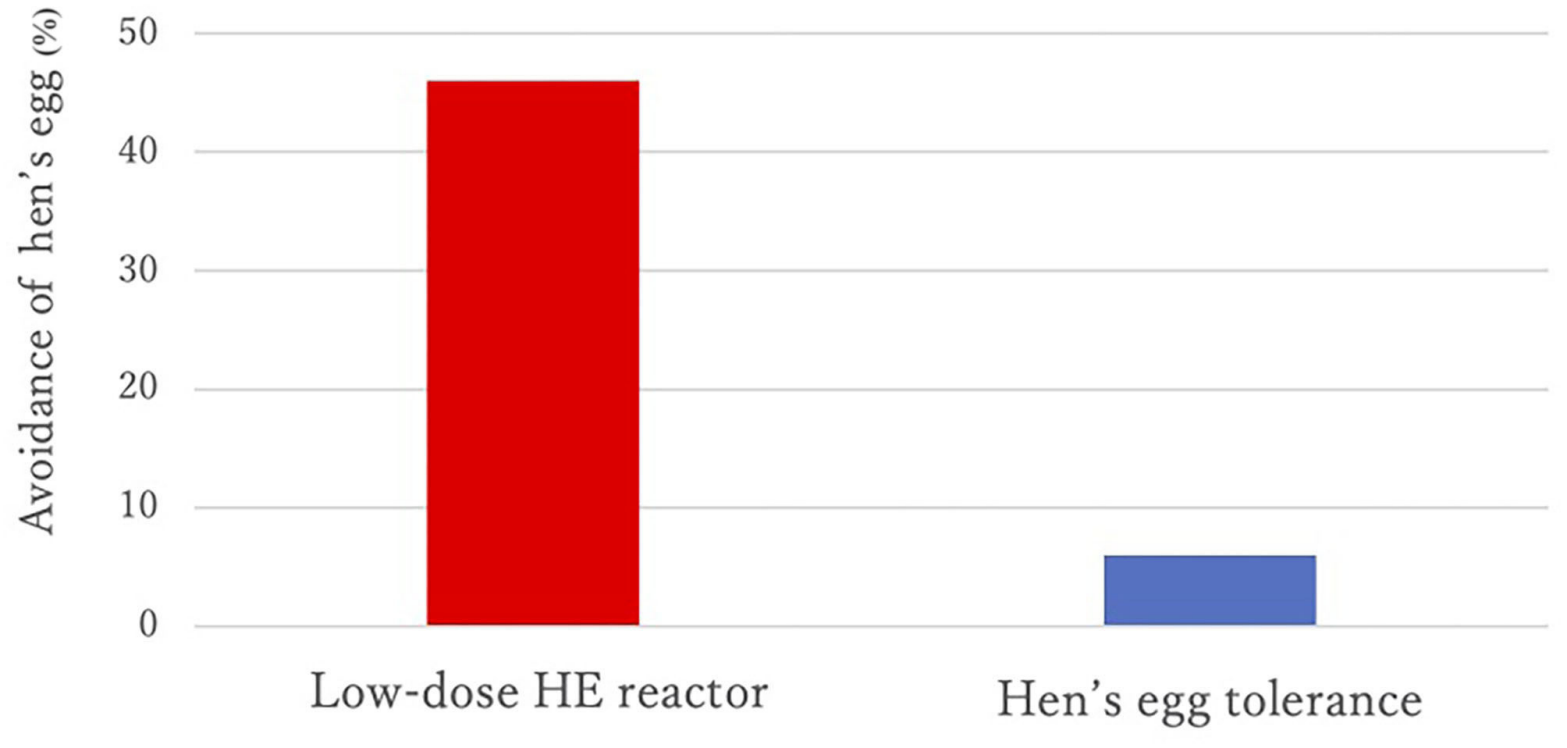
Avoidance of hen's egg of the two groups.

Sex, prevalence of atopic dermatitis, prevalence of allergic rhinitis, and total IgE values at <2 years of age were not different between the two groups, but the prevalence of asthma was significantly higher in the HE tolerance group than in the low-dose HE reactor group (see Table 1). All children were sensitized to HE before 2 years of age. A lower proportion of strict avoidance of HE was observed in the HE tolerance group than in the low-dose HE reactor group (6 vs. 46%, *p* = 0.006, see Figure 2). OM-sIgE levels in children younger than 2 years were significantly higher in the low-dose HE reactor group than in the HE tolerance group (median [interquartile], 26.7 UA/mL [11.9–53.4] vs. 7.9 UA/mL [0.35–23.4]; *p* = 0.024, see Table 1). The peak egg white IgE levels >50 UA/ml were significantly higher in the low-dose HE reactor group than in the HE tolerance group (75 vs. 25%). Although OM-sIgE levels at <2 years of age and the peak egg white IgE levels >50 UA/ml in the low-dose HE reactor group were significantly higher than that in the HE tolerance group, multivariate logistic regression analysis revealed that avoidance of HE was associated with low-dose HE reactor even after adjusting the OM-sIgE levels at <2 years of age or the peak egg white IgE levels >50 UA/ml, respectively (adjusted odds ratio (aOR) = 14.5; 95% confidence interval (CI): 1.63–128.7, aOR = 10.8; 95%CI: 1.16–99.4, respectively, see Table 2).

**Table 1:** Clinical features of the two groups.

		**Low-dose hen's egg reactor (*n =* 26)**	**Hen's egg tolerance (*n* = 17)**	***p*-value**
Male	*n* (%)	15 (58)	14 (82)	0.111
**Allergic history**				
Atopic dermatitis	*n* (%)	22 (85)	12 (71)	0.445
Asthma	*n* (%)	**17 (65)**	**17 (100)**	**0.007**
Allergic rhinitis	*n* (%)	6 (23)	8 (47)	0.182
Total IgE levels at less than 2 years of age	(IU/mL)^*^	271.5 (180.5-803.2)	234.0 (89.9–1,081)	0.777
**Specific IgE levels at** **<** **2 years of age**				
Egg white	(UA/mL)^*^	58.1 (33.9–94.1)	33.0 (20.4–80.2)	0.284
Ovomucoid	(UA/mL)^*^	**26.7 (11.9–53.4)**	**7.9 (0.35–23.4)**	**0.024**
Peak egg white IgE levels >50 UA/ml	*n* (%)	**21 (75)**	**7 (25)**	**0.011**
Introduction age	(y)^*^	4 (3.75–5)	4 (3,4)	0.097

*Fisher's exact test and Mann Whitney-U-test*.

* *median (interquartile range)*.

*Bold values indicate p < 0.05*.

**Table 2:** Multiple logistic regression models: Adjusted Odds of low-dose hen's egg reactor at age 6 years. (A) avoidance of HE and ovomucoid-specific IgE levels at <2 years of age, (B) peak egg white IgE levels >50 UA/ml and avoidance of hen's egg.

**Factors of low-dose hen's egg reactor at age 6 years**	adjusted^*^ odds ratio	**95% confidence interval**
**(A)**		
Avoidance of hen's egg	14.5	1.63–128.7
**(B)**		
Avoidance of hen's egg	10.8	1.17 – 99.4

^*^Ovomucoid-specific IgE levels were adjusted.

^*^Peak egg white IgE levels >50 UA/ml or avoidance of hen's egg were adjusted.

## Discussion

Our study provided evidence that a higher proportion of experiencing strict avoidance was observed in school-aged children with low-dose HE reactor than in those who achieved HE tolerance. Our findings also showed that strict avoidance of HE intake for a long period from early infancy might increase the risk of a persistent HE allergy in school-going children.

Our results show that although OM-sIgE levels in children younger than 2 years and the peak egg white IgE levels >50 UA/ml were significantly higher in the low-dose HE reactor group than in the HE tolerance group, multivariate logistic regression analysis revealed that avoidance of HE was associated with low-dose HE reactor even after adjusting the OM-sIgE levels at <2 years of age or the peak egg white IgE levels >50 UA/ml, respectively. Our findings are consistent with the previous study revealed that patients with an egg white-specific IgE (EW-sIgE) level of >50 kU/L increased the risk of low-dose HE reactor in school-age children (3). Despite high EW- and OM-sIgE levels, continuous avoidance for heated HE may increase the risk of low-dose HE reactor.

To the best of our knowledge, this study is the first to demonstrate the risk of strict avoidance of HE for a long period in allergic children in a real-world setting. We used heated HEW, which was similar to baked egg, instead of raw HE in our study. Baked egg tolerance was previously observed in 64–80% of children with HE allergies (11, 12). However, in our study, children with HE allergy could not tolerate even small doses of heated HEW, which means they also could not tolerate baked cakes and biscuits containing HE.

The strength of this study is that the HE allergy outcomes were confirmed by the gold standard method of OFC. In Japan, majority of children tend to undergo OFCs at 6 years of age before being elementary schoolers. Age of 6 years is very important point for food allergy patients and their families because elementary schools in Japan provide mandatory school lunch for children. As mentioned earlier, our previous RCT (PETIT Study) demonstrated that early introduction of HE (starting from a very small dose administered in two steps) was effective in preventing HE allergy development at 12 months of age, although 80% of participants at 6 months of age were already sensitized to HE. Our study results were consistent with the PETIT study (6) since HE tolerance group who were already sensitized to HE in young infants could achieve oral tolerance compared to the low-dose HE reactor group by eating HE without avoidance of HE until the age of 6 years.

The European Academy of Allergy and Clinical Immunology (EAACI) guidelines (13) state that OIT is a viable treatment option to increase the threshold of reaction in children with persistent HE allergy from 4 to 5 years of age. However, we would like to propose that low-dose OIT starting with small amounts of allergenic foods may be the best and safest management strategy for all HE-allergic infants to induce oral tolerance for HE. Further studies using the same strategy in practice are required to strengthen our study results.

There are two limitations to this study. First, this study was an observational, therefore, its evidence level is lower than that of an RCT. Second, this study included a case from a single facility.

In summary, our study showed that strict avoidance of HE intake for a long period from early infancy increased the risk of low-dose HE reactor in school-age children. We reiterate that the recommendation to avoid an allergenic food item should not be decided based on a simple finding of IgE sensitization. Even in children who are already sensitized and have developed an HE allergy, we believe that introduction of low-dose HE might resolve HE allergy as a safe method of OIT. Our findings significantly highlight the risk of avoidance of HE with children with HE allergy by high EW- and OM-sIgE levels.

## Data Availability Statement

The datasets presented in this article are not readily available due to ethical considerations and the privacy laws of Japan.

## Ethics Statement

The studies involving human participants were reviewed and approved by the ethics committee of the National Center for Child Health and Development (2019-066). Written informed consent from the participants' legal guardian/next of kin was not required to participate in this study in accordance with the national legislation and the institutional requirements.

## Author Contributions

YMiyag managed and analyzed the data. KY-H and YO obtained the funding [Grant-in-Aid for Scientific Research (B) 19H03569], contributed to the conception and design of the study, and interpretation and discussion of the results. All authors read and approved the final manuscript.

## Conflict of Interest

The authors declare that the research was conducted in the absence of any commercial or financial relationships that could be construed as a potential conflict of interest.
